# Transforming patterned defects into dynamic poly-regional topographies in liquid crystal oligomers†

**DOI:** 10.1039/d4mh00131a

**Published:** 2024-04-19

**Authors:** Yuxin You, Youssef M. Golestani, Dirk J. Broer, Tinghong Yang, Guofu Zhou, Robin L. B. Selinger, Dong Yuan, Danqing Liu

**Affiliations:** a Joint Research Lab of Devices Integrated Responsive Materials (DIRM), South China Normal University Guangzhou 510006 China dong.yuan@guohua-oet.com; b Human Interactive Materials (HIM), Department of Chemical Engineering and Chemistry, Eindhoven University of Technology Groene Loper 3, Eindhoven 5612AE The Netherlands d.liu1@tue.nl; c Institute for Complex Molecular Systems (ICMS), Eindhoven University of Technology Groene Loper 3, Eindhoven 5612AE The Netherlands; d Department of Physics, Kent State University Kent OH 44242 USA rselinge@kent.edu; e Advanced Materials and Liquid Crystal Institute, Kent State University Kent OH 44242 USA

## Abstract

We create high-aspect-ratio dynamic poly-regional surface topographies in a coating of a main-chain liquid crystal oligomer network (LCON). The topographies form at the topological defects in the director pattern organized in an array which are controlled by photopatterning of the alignment layer. The defect regions are activated by heat and/or light irradiation to form reversible topographic structures. Intrinsically, the LCON is rubbery and sensitive to temperature changes, resulting in shape transformations. We further advanced such system to make it light-responsive by incorporating azobenzene moieties. Actuation reduces the molecular order of the LCON coating that remains firmly adhered to the substrate which gives directional shear stresses around the topological defects. The stresses relax by deforming the surfaces by forming elevations or indents, depending on the type of defects. The formed topographies exhibit various features, including two types of protrusions, ridges and valleys. These poly-regional structures exhibit a large modulation amplitude of close to 60%, which is 6 times larger than the ones formed in liquid crystal networks (LCNs). After cooling or by blue light irradiation, the topographies are erased to the initial flat surface. A finite element method (FEM) model is adopted to simulate structures of surface topographies. These dynamic surface topographies with multilevel textures and large amplitude expand the application range, from haptics, controlled cell growth, to intelligent surfaces with adjustable adhesion and tribology.

## Abstract 2

We transform patterned defects into high-aspect-ratio poly-regional surface topographies. The active intricate textures are induced by generated shear forces under external stimuli.

## Graphical abstract

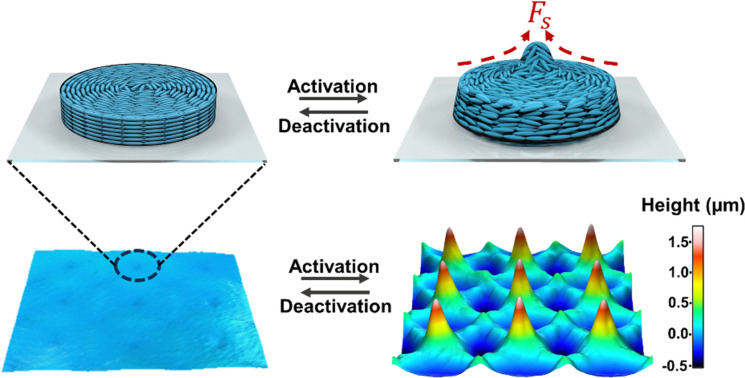

**New concepts**

Our work introduces a new concept by transforming patterned defects into high-aspect-ratio poly-regional surface topographies. This innovative approach involves inducing topographies at multiple topological defects, organized through patterning of the crosslinkable liquid crystal oligomers. Unlike existing research that uses conventional liquid crystal networks (LCNs) to boost surface morphing through free volume generation, we take advantage of the anisotropic deformation properties of liquid crystal oligomer networks (LCONs) to generate directional shear stresses around the defects, thereby creating large, intricate textures. By applying thermal and light individually or in combination, these dynamic surface topographies exhibit a large modulation amplitude, nearly 60%, which is significantly higher than that of LCNs, typically reported to be around 10%. The experimental work combined with the numerical simulations demonstrate remarkable agreement. We anticipate our results will generate great interest among scientists working in the field of stimuli-responsive materials, nano/micro-actuators and smart surfaces.

## Introduction

In nature, surface topographies play an important role in survival and reproduction of living creatures. For instance, octopuses modulate their epidermis from smooth to rough and spiky to enhance friction and adapt to their surroundings.^1,2^ Cuttlefish expand or contract their pigmented skin to generate a rich array of coordinated textures on their surface when they need to camouflage or communicate.^3^ Mammalian sperm respond to changeable microstructures (*e.g.*, microvilli and folds) on the female reproductive tract and alter their strategy accordingly during the migration.^4^ The ingenuity of these natural examples lies in regulating the functionality of the surface by switching their topographies in order to interact with the surrounding environment. Among those, the most interesting structures are found on cuttlefish skin, which exhibit various sizes and shapes, such as conical papillae, reef-like protrusion, high ridges, and spikey horns.^5,6^ These multiple structures occur concurrently in distinct areas, creating a surface that resembles seaweed, a pile of rocks, or even a sandy hill. In this case, the surface functionality is largely influenced by the variety of structures. Learning from nature, scientists have developed various dynamic surfaces,^7–12^ however, those surfaces are limited in a modulated transition from flat to singular texture, limiting their applications. Poly-regional surface topographies with complex structures are still challenging and anticipated to benefit many new functions in the fields of haptics, biomedicine, and microfluidic manipulation.

Many efforts have been devoted to producing dynamic surfaces through developing both materials and new fabrication strategies.^13–15^ Some of the most interesting materials are liquid crystal polymer networks (LCNs), typically produced by photo-polymerization of liquid crystal diacrylate monomers. The molecular configurations of those monomers can be established by using the existing liquid crystal display technology. The established alignments can be further captured into polymer networks through photo-polymerization.^16–18^ The degree of the molecular orientation is characterized by the scalar order parameter, *S*, which describes how well the mesogenic units are aligned toward a direction *n*, defined as director.^19^ When subjected to external stimuli (*e.g.*, heat, light illumination, or/and electric fields), *S* decreases, disturbing the polymeric chains. This leads to anisotropic deformations manifested as contraction along the director and expansion perpendicular to the director.^20,21^ Simultaneously, extra voids between molecules are created (referred to as free volume). Built upon this, one of the methods to create surface topographies on a confined LCN is based on the free volume generation.^10,22^ Due to the restriction from the substrate, the lateral deformations are largely prohibited and the extra volume can only escape at the coating surfaces to form the topographies. The formed dynamic surface topographies have shown new applications such as self-cleaning action,^23^ and controlled adhesion and release.^24^ Due to the limitation of the relatively high crosslink density and the glassy networks, the typical topographic modulation in LCN is approximately 10% with respect to the initial coating thickness,^10,22–26^ which is not large enough for many applications.

Unlike LCNs, which create surface topographies through free volume generation,^22^ in this work, we investigate the use of the anisotropic deformation properties to achieve both high actuation strain and complex surface topographies in a coating. For this purpose, we develop liquid crystal oligomer networks (LCONs) by crosslinking the liquid crystal oligomers. Architecturally, the LCONs are of the main chain type, with the liquid crystal mesogens integrated into the backbone of the oligomer main chains.^27,28^ Mechanically, LCONs possess glass transition temperature (*T*_g_) far below room temperature at around −20 °C which allows shear forces to take place during the deformations.^29,30^ Intrinsically, LCONs undergo phase transitions, and thus, *S* is reduced significantly at elevated temperatures.^31^ Moreover, by incorporating azobenzene derivatives, a photo-mechanical response is obtained.^32–38^ These two triggers can be applied individually, yet they also can be applied simultaneously to achieve a synergistic effect which further enhances the amplitude of deformation. In order to form complex poly-regional topographic structures, we introduce patterned defect arrays.^39,40^ These arrays are written into the liquid crystal oligomers by photo-alignment technology based on the digital micromirror device (DMD).^41^ The resulting topographies possess several morphological features, including two types of protrusions with different heights, a ridge connecting them, and a singular indentation. These topographic surfaces exhibit a large modulation height, reaching up to 2.9 μm for a coating with a thickness of 5 μm, corresponding to an amplitude of close to 60%. The magnitude of the topographical deformation produced by this method is 6 times larger than the ones developed in LCNs.^10,22–26^

## Results and discussion

**Fig. 1:**
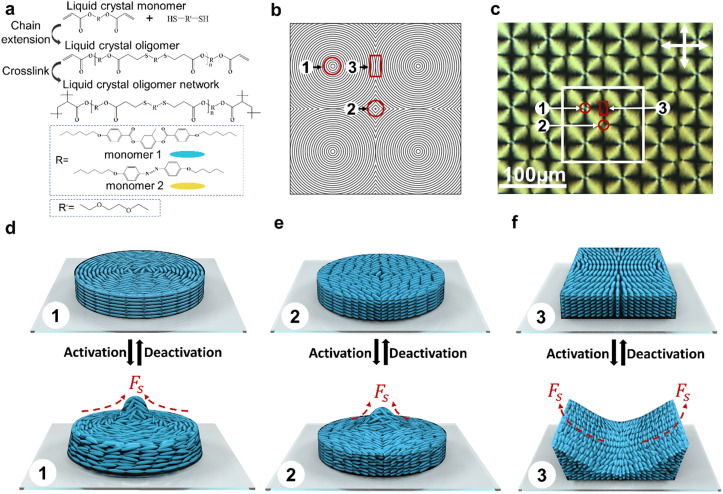
Principle of the formation of surface topographies. (a) Schematic of the two-step synthesis of LCONs. (b) Top view of the molecular alignment. (c) POM image of the patterned LCON coating. The labeled regions correspond to the regions shown in (b). 3D schematics of the molecular alignment before actuation, and the resultant deformation after actuation: region 1 (d), region 2 (e), and region 3 (f).

We prepared the main-chain LCON coatings containing preprogrammed information by a two-step synthesis process. First, a Michael addition reaction between the diacrylate mesogen/azobenzene derivative and the flexible dithiol spacer was adopted to synthesize the acrylate-terminated oligomers (Fig. 1a). In this step, we tuned the degree of oligomerization of the oligomers by adjusting the stoichiometric ratio between acrylate and thiol to achieve the optimal viscosity for liquid crystal oligomers to follow the alignment. Moreover, we modulated the concentrations of azobenzene to explore their effect on the photomechanical properties of LCON. The degree of oligomerization calculated from the ^1^H NMR spectrum of the oligomers suggests the chain length *n* is around 8.5 (Fig. S1, ESI†). These oligomers were spin-coated on the substrate with a patterned photo-alignment layer, established by using a DMD. For this paper, we select patterns of +1 defects with azimuthal orientation and each of them is arranged into square pocket arrays. Based on this, we obtain complex defect arrays that contain three distinguished regions. Region 1 is the area encapsulated by the +1 defects. Each four +1 defects meet in a center, forming a −1 defect (region 2), while two adjacent defects meet at an intersection, forming region 3 (Fig. 1b). Subsequently, the patterned oligomer coating was photopolymerized *via* free-radical reaction at the nematic phase to form networks (Fig. S2, ESI†). During this process, a green light was used to avoid premature *trans* to *cis* isomerization of azobenzene. The LCON coating with patterned alignment is confirmed by the polarized optical microscope (POM), as shown in Fig. 1c, which also features three labeled regions, corresponding to the positions shown in Fig. 1b. The schematics of the molecular orientation of each type of region and their deformation are shown in Fig. 1d–f. The LCON coating is confined on a rigid substrate. The underlying mechanism of dimensional changes on the surface is based on generating the shear forces (*F*_s_), caused by the molecular order reduction,^33,42^*via* heat and/or light illumination. This principle is schematically illustrated for regions 1–3, respectively. For region 1, the generated shear forces direct towards the core of the +1 defect with azimuthal orientation, moving the material from the periphery to the center, leading to a protrusion emerging (Fig. 1d). The molecular orientation in region 2 is close to a square, a reminiscent of bend geometry, and the resultant force is towards the defect center, forming a protrusion as well (Fig. 1e). Region 3 is composed of splay and bend distortions. Consequently, the surface indents in the middle, where the director has splay; and elevates at the sides, where the director is bent. The generated shear forces direct in the opposite direction, pushing the material away from the center, as shown schematically in Fig. 1f.

**Fig. 2:**
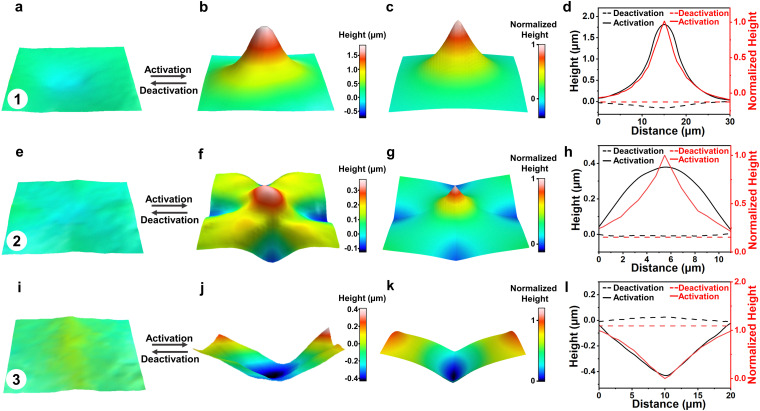
Formation of multiple topographies. 3D images of the initial and actuated states measured by 3D profilometer: (a) and (b) region 1; (e) and (f) region 2; (i) and (j) region 3. (c), (g) and (k) Corresponding simulations, obtained from actuating a flat nematic elastomer coating with the director field design shown in Fig. S7, ESI.† (d), (h) and (l) Cross-section surface profiles with experiment in black and simulation in red for each region. Dashed and solids lines are initial and actuated states, respectively.

The corresponding surface topographies of LCON coating for each region are mapped by a 3D profilometer (Methods). Initially, at a nematic phase around 50 °C, the LCON coating exhibited a flat surface with minor surface reliefs observed most likely due to the polymerized shrinkage.^23^ Before actuation, we brought the sample to room temperature (RT) and we observed that the surface was slightly indented in region 1 and 2, and protruded in region 3 (Fig. 2a, e and i). Cooling to RT increased *S*, giving rise to an inverse behavior. At elevated temperatures, *S* decreased, regions 1 and 2 were protruded, and region 3 was indented in the middle, as shown in Fig. 2b, f and j. Discussed in the method section, we model the shape transformation of surfaces, using finite element method (FEM) simulations. Fig. 2c, g and k are the simulation results for regions 1, 2, and 3, respectively. Our computational results confirm the shape and the symmetry of the structures that appear in the regions. For instance, for the −1 defect in region 2 with four splay and four bend sections in the director field, the final topography develops four ridges and four valleys, as shown in Fig. 2f and g. Fig. 2d, h and l present the cross-section surface profiles of each region, with experimental data in black and computational results in red. The height of the formed structure of region 1 reaches 2 μm, while that of region 2 is 0.35 μm. This difference can be understood by looking at the difference in their director fields: a +1 defect with azimuthal orientation comprises of pure bend, and a −1 defect contains both bend and splay in the director field, which leads to the shear force being weaker. The deformation for region 3 is −0.5 μm, as it indents, due to the rich splay in the director field. The simulations show similar profiles to the experiments, noting that the slight discrepancy between the experiments and the simulations possibly stems from the limited resolution of molecular alignment, as compared to the simulations. Once the stimulus is removed, both protrusions and depressions can be erased by the elastic retractive force of the network,^43^ bringing the system to the original state. This reversibility was realized by cooling down to RT.

**Fig. 3:**
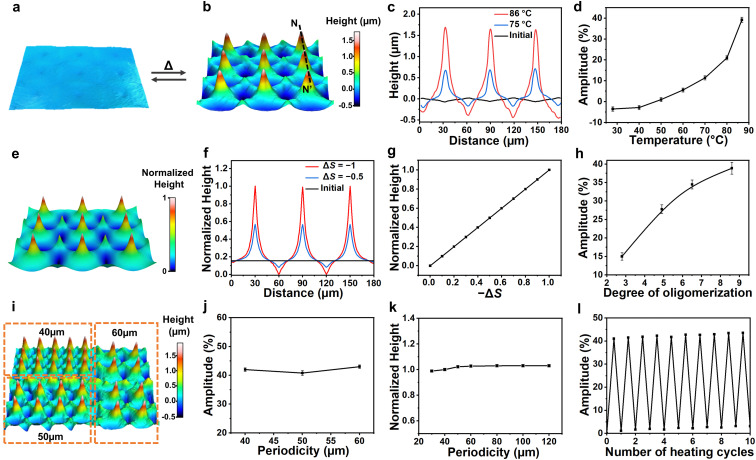
Thermal-responsive poly-regional surfaces. (a) 3D visualization of the surface at the initial state, and (b) actuated state upon heating. (c) Cross-section surface profiles along line NN′. (d) Influence of temperature on deformation amplitude. (e) 3D visualization of the surface from simulations. (f) Cross-section surface profiles from simulations. (g) Simulation study of the dependency of the deformation on *S*. (h) Influence of the degree of oligomerization on the amplitude of thermal response. (i) 3D visualization of an actuated LCON coating with different periodicity. Plots of deformation as a function of periodicity: (j) experiment, (k) simulation. (l) Repeated cycles for the thermal response of the coatings. The experimental results shown in this figure are from the samples containing 15 wt% azobenzene.

After analyzing each region individually, we examined the defect arrays, with different experimental conditions and computational parametrization. Upon heating, the surface deformed and shown row-upon-row multiple structures, as shown in Fig. 3a and b. Fig. 3c presents the cross-section surface profiles along line NN′. The height difference between valleys and peaks reaches a value of approximately 2 μm, giving the modulation amplitude, which is defined by the height difference divided by the coating thickness, of 40%. The influence of temperature on deformation amplitude is shown in Fig. 3d. The result shows that the amplitude increases exponentially by increasing the temperature, which substantially reduces *S* as it approaches the nematic to isotropic phase transition (Fig. S3, ESI†). Fig. 3e, shows the simulation results of the surface topographies for a 3 × 3 array of defects after the *S* has been decreased by 1, Δ*S* = −1. In Fig. 3f, we plot the cross-section profiles in the simulation by intersecting Fig. 3e with the plane that passed through the cores of +1 defects. It can be seen that the experimental and computational studies provide almost identical results for 3D topographies, cross-section profiles, as well as the location of the extremums. While our data supports that the amplitude of deformations increases as we increase the temperature, *i.e.*, decreasing *S*, such dependency is different between experiment and computation (Fig. 3d and g). Based on the Hamiltonian that we modeled here (Methods), the deformations increase linearly with *S*, which is not the case in the experiment shown in Fig. 3d. The reason might be related to the temperature dependency of the storage modulus in the experiment,^44,45^ while in the model, we assume that the mechanical properties do not change during thermal actuation.

We further investigated a number of experimental parameters that affect the thermal-responsive deformations. We observed that the degree of oligomerization influences the modulation amplitude. As shown in Fig. 3h, the amplitude increases with increasing degree of oligomerization which leads to a lower crosslink density, and thus, lowers the storage modulus.^45^ Subsequently, materials are easier to shear. We also studied the influence of the periodicity of the structure which is defined as the center-to-center distance of a pair of adjacent +1 defects, see Fig. 3i. Both our experiment (Fig. 3j) and simulation (Fig. 3k) results indicate that there is no significant impact of periodicity on the amplitude of the deformation. Furthermore, we varied the coating thickness, as shown in Fig. S5, ESI,† and we found that the thicker coating deforms greater. This is interpreted as increased thickness providing more materials for shearing. Moreover, our samples can cycle many times by heating and cooling with no obvious degradation of the thermal actuation, as shown in Fig. 3l.

When the actuation temperature is above 100 °C, an interesting phenomenon occurs where permanent surface structures are produced. Typically, at 120 °C, the amplitude of the deformation is 80% (Fig. S4, ESI†). This phenomenon is most likely attributed to yield stress, which is the stress beyond the elastic limit, the material undergoes plastic deformation and cannot be fully recovered after the external force is withdrawn.^46,47^

**Fig. 4:**
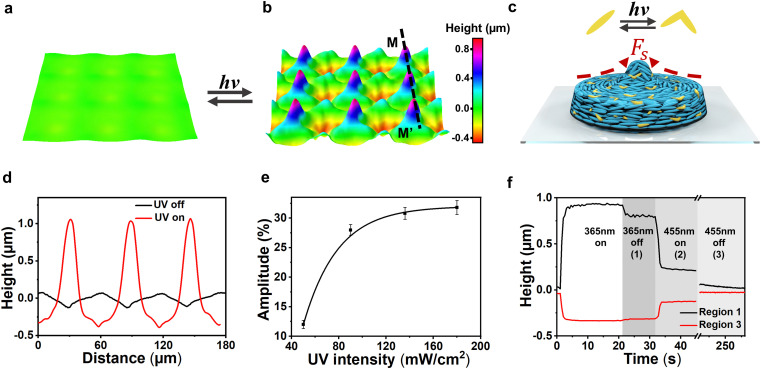
Photo-responsive poly-regional surfaces. 3D visualization of the surface at the (a) initial state, and (b) actuated state upon UV illumination. The actuating light intensity is 90 mW cm^−2^. The deformation is reversible upon blue light illumination and a heat release. (c) Schematic illustration of the deformation at region 1 actuated by UV illumination, the embedded azobenzene isomerizes from *trans* to *cis*. (d) The corresponding profiles along line MM’. (e) Influence of UV intensity on the deformation amplitude. (f) Kinetics of the deformation and the recovery. The sample contains 15 wt% azobenzene.

Next, we studied the photo-responsiveness of poly-regional surface topographies which are monitored *in situ* by digital holographic microscope (DHM). Upon UV light irradiation at 365 nm, the surface protruded and indented immediately, as shown in Fig. 4a and b. During UV illumination, the azobenzene embedded in the backbone absorbs UV light and isomerizes from *trans* to *cis*, deforming the main chains of the network (Fig. 4c). Similar to actuation by heat, this process also leads to a decrease in *S*, which further produces topographic structures at the topological regions. Fig. 4d presents the cross-section surface profiles along line MM’. The difference in height between the valleys and the hills reaches a value of 1.35 μm, which corresponds to a modulation amplitude of 28% with respect to a coating thickness of 4.8 μm. This value is lower compared to the case of thermo-responsive actuation. We note here that the mechanism that contributes to such difference may be due to the nonuniform actuation of the sample under UV irradiation. While heating increases the temperature almost uniformly through the thickness of the coating, UV intensity is significantly different on the top and bottom of the coating. We model so, by adding gradient to the actuation in our simulation, see Fig. S9, ESI.†

Experimentally, the influence of UV light intensity is shown in Fig. 4e. With increasing UV light intensity, the deformation increases as the azobenzene reaches a higher photo-stationary state. We also investigated the light-induced thermal effect. The coating temperature was raised to 32 °C during UV exposure with the light intensity of 90 mW cm^−2^. This temperature corresponds to a thermally actuated deformation amplitude of less than 1%, which implies the temperature effect is ignorable. When the UV light was switched off, the relaxation process experienced three phases, as shown in Fig. 4f. In phase 1, the *cis* populating was stopping, the deformation rapidly decreased by 15%. In phase 2, the blue light (455 nm) was switched on to promote relaxation, during which 60% of the deformed surface was erased in 1.5 s. Finally, in phase 3, the heating process was applied to enhance the mobility of the network to facilitate a full recovery of 100% at RT.

**Fig. 5:**
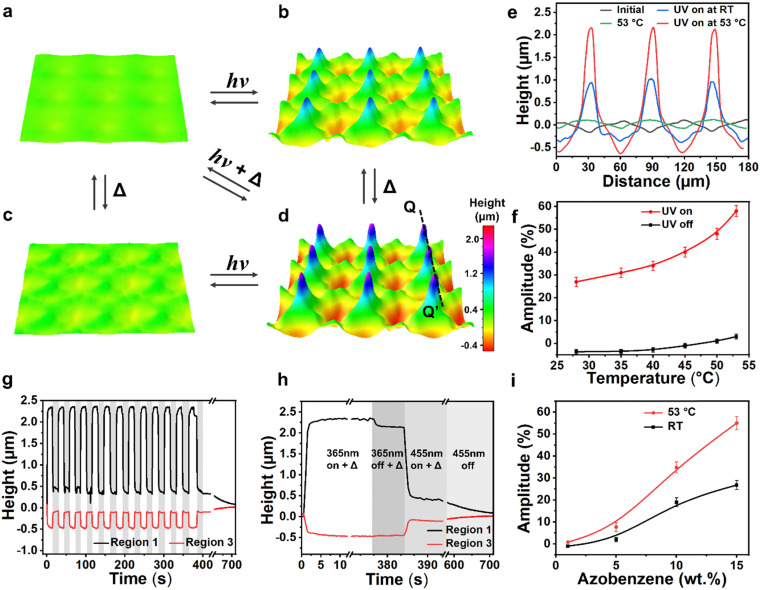
UV light and heat synergistically actuated surface topographies. 3D visualization of the surface at (a) the initial state at RT, (b) during UV illumination, (c) heating to 53 °C and (d) UV illumination at 53 °C. The deformation is reversible upon blue light illumination and a heat release. (e) The corresponding cross-section profiles of surface topographies along line QQ’ at the initial and actuated state. (f) Influence of temperature on the deformation amplitude when UV light is switched on and off. (g) Repeated cycles for the UV response at 53 °C. (h) Kinetics of the deformation and the recovery (*Δ* = 53 °C). The sample for (a)–(h) contains 15 wt% azobenzene with a coating thickness of around 4.8 μm. (i) Influence of azobenzene concentration on the deformation amplitude induced by light illumination at RT and 53 °C.

Apart from individual thermal and photo-induced deformations, we explored dual responsiveness by applying thermal and light illumination simultaneously. The dynamic deformation process was measured by DHM. The 3D visualization is given in Fig. 5a–d, and Video S1, ESI.† First, we exposed the surface to UV light at RT, the amplitude of deformation is typically 28%. With UV light on, we applied heat, upon heating to 53 °C, the deformation amplitude increased to 58%. By heating alone at 53 °C, the deformation amplitude only reaches 4%. The corresponding surface profiles along line QQ′ are shown in Fig. 5e. We further compared the deformation at varied temperatures with UV light on and off, as shown in Fig. 5f. We can see that the synergistic effect of heat and light is almost twice the sum of the individual one. We explain this significant deformation at higher temperatures by considering the enhancement of the mobility of the main chains. And thus, the materials are easier to shear under UV illumination. This process is reversible when keeping the temperature constant at 53 °C while switching the UV light on and off (Fig. 5g). Similar to the relaxation of photo-response, when switching off the UV light, there are three phases to reach full recovery, as shown in Fig. 5h.

To reveal the relationships between the deformation and azobenzene concentration, we performed experiments with 1 wt%, 5 wt%, 10 wt%, and 15 wt% of azobenzene. While keeping the UV light on at 53 °C, their deformation amplitude is 0%, 7%, 36%, and 58%, respectively (Fig. 5i). This result is interpreted as the higher the concentration, the more azobenzene molecules bend to their *cis* state, giving rise to disorder more aligned molecules. We further investigated the influence of coating thickness on the deformation (Fig. S6, ESI†). Results show that at 53 °C, even at a thickness of only 2 μm, the topographies exhibit a large deformation amplitude. As the thickness increases, the deformations show a notable increase, indicating a more significant synergistic effect, allowing more materials to shear more readily.

Similar to thermal-responsive deformations, here, we also observed that permanent deformation occurs when the coating is heated above 60 °C while exposed to UV light. In this case, azobenzene groups tend to rotate out of the plane of the coating to occupy a more stable topological position at their *cis* state. This realignment of azobenzene at elevated temperatures is allowed due to the rubbery material system, which sufficiently provides reorientation mobility.^48^ The initial order state cannot be retained even by the blue light illumination, due to the interaction between the electromagnetic field of the propagating light toward the azobenzene being less, thus causing permanent deformation.

## Conclusions

In conclusion, we have demonstrated a preprogrammable LCON coating with active surface topographies, especially those exhibiting poly-regional deformations such as protrusions, ridges, and depressions controlled by inscribed director field patterns. These active surfaces are triggered by thermal and light individually and combined. When activated, the deformations induced by one trigger can be enhanced by adding the other one. The formed topographies present a large modulation amplitude of nearly 60%, which is 6 times larger than that of LCNs. This is made possible by the soft elastic properties of LCONs, which allow the shear forces to introduce anisotropic deformations in a confined LCON. These shear forces push the material to move and produce remarkable dimensional changes on the surface. The process is reversible, as the elastic retractive force of the network brings the surface back to its original state. Furthermore, an FEM elastodynamic model is developed to justify the shape of the poly-regional features and support our experimental results. We anticipate that these poly-regional dynamic surface topographies can be used for a variety of applications, not only in robotic handling which is controlled by switchable friction, but also in drug delivery and tissue engineering, to promote the growth, proliferation, and differentiation of cells.

## Methods

### Experimental materials

The Monomer 1 (RM82, 1,4-bis-[4-(6-acryloyloxyhexyloxy)-benzoyloxy]-2-methylbenzene) was purchased from Jiangsu Hecheng Advanced Materials Co., Ltd. The photoresponsive monomer 2 (4,4′-bis(6-acryloyloxyhexyloxy)azobenzene) was purchased from Synthon. For the control groups, the amount of monomer 2 was changed to 1 wt%, 5 wt%, 10 wt%, and 15 wt% respectively. To keep the ratio of crosslinkers to be the same, the concentration of monomer 1 was correspondingly changed. The dithiol chain extender (3,6-dioxa-1,8-octanedithiol) was obtained from Sigma Aldrich. The molar ratios between thiol and acrylate were tuned to obtain different degrees of oligomerization. The catalyst dipropylamine (DPA) was obtained from Shanghai Macklin Biochemical Co., Ltd. The photoinitiator 784 was obtained from Ryoji chem's branch office in China. The photo-alignment agent, PAAD-22, was obtained from Beam Co. (USA). In this experiment, all the chemicals were used directly and without further purification.

### Preparation of the patterned LCON coatings

Glass substrates were first cleaned with acetone and isopropanol *via* an ultrasonic water bath for 20 min, respectively. Then the glasses were dried up and put in UV-ozone for 20 min for surface treatment. The photo-alignment agent PAAD-22 was diluted 3 times with *N*,*N*-dimethylformamide (DMF) and then spin-coated on the glass substrate at the speed of 800 rpm for 10 s followed by 3000 rpm for 40 s. Subsequently, the coated glass substrate was baked at 100 °C for 10 minutes to completely evaporate the DMF to obtain a photo-alignment layer on the glass substrate. A digital micro-mirror-based dynamic microlithography system was used to inscribe the alignment patterns on the coated substrate. To fabricate the LCON coating, we first synthesized the liquid crystal oligomers *via* a base-catalyzed thiol-acrylate Michael addition reaction. Monomer 1, monomer 2, dithiol, DPA, and photoinitiator were dissolved in the dichloromethane solvent. The mixture was stirred overnight at RT. After the Michael addition reaction was completed, the formed oligomers dissolved in dichloromethane solution (5 wt%) were spin-coated at 1000 rpm (acceleration rate 500 rpm s^−1^) for 8 s on the patterned substrate and then placed on a hot stage around 55 °C to evaporate the solvent. Lastly, the LCON coating was obtained after 1 hour of polymerization using a 530 nm LED lamp (Thorlabs).

### Characterization

The optical properties of the LCON coatings were analyzed using a polarized optical microscope (Leica DM2700P) equipped with a thermo-controlled stage (Linkam THMS600). The room temperature depicted in this paper is around 26 °C. The phase transition temperatures were confirmed using differential scanning calorimetry (Mettler Toledo). The thickness of the coatings and the surface topographies were measured by Leica 3D profilometer in confocal mode (Leica DCM8). The photo-responsive surface topographies were measured by a digital holography microscope (Lyncée Tec.). The photo response was actuated with a UV lamp (*λ* = 365 nm) and a blue lamp (*λ* = 455 nm) (Thorlabs) at 90 mW cm^−2^ and 50 mW cm^−2^, respectively.

### Finite element

We model the actuation of nematic elastomer thin film coatings discretized in a structured tetrahedral mesh^49^ with physical dimensions of 180 × 180 × 5 μm^3^. We assume that the stimuli-driven mechanical response has a Hamiltonian of the form:
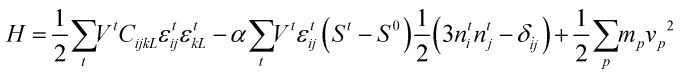
where the first term is the elastic strain energy of the sample summed over all elements in the tetrahedral mesh, 
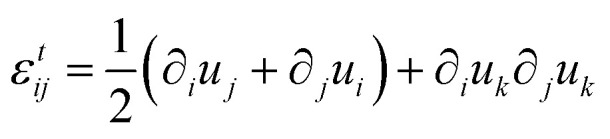
, is the Green–Lagrange strain, *C*_*ijkL*_ is the tensor of elastic moduli, *V*^*t*^ is the volume of each tetrahedron in the initial reference state. The second term is the potential energy associated with the coupling of strain and nematic order tensor, where *α* is the coupling coefficient. *S*^*t*^ is the local nematic scalar order parameter in volume element *t* at a given time step, and *S*^0^ is the value of *S* at the beginning of the simulation and changes linearly with time. *S* is either spatially uniform through the thickness, in case of actuation by heat, or with a gradient, in case of actuation by UV.

To enable the system to reach mechanical equilibrium, node velocities, *ν*_p_, are reduced by a damping factor of 0.998 at each time step to gradually reduce the kinetic energy and relax to the minimum of the potential energy. We monitor the system's potential energy to ensure that it has reached mechanical equilibrium. The material density was set at 1.2 g cm^−3^ in accordance with the experimental measurements for a similar material^50^ and the lumped-mass approximation divides each tetrahedral volume element's mass, *m*_p_, evenly among its four nodes, p.

To each volume element, we assign a local nematic director orientation which is the director orientation of material after it was cross-linked. We also assume that the cross-link density of this polymer material is high enough that the nematic director does not reorient under mechanical stress. Therefore, the nematic director is defined at the time of cross-linking and remains constant in the body frame. Following the design in the experiment, our model contains a square lattice of +1 defect lines extending from the bottom of the coating to the top and oriented perpendicular to the rigid substrate. We construct a 2D director field with a 3 × 3 array of topological defects, as shown in Fig. S7, ESI.† Similar to experimental design, each defect is located in the center of a square mosaic tile where the director at a given position (*x*,*y*) is 
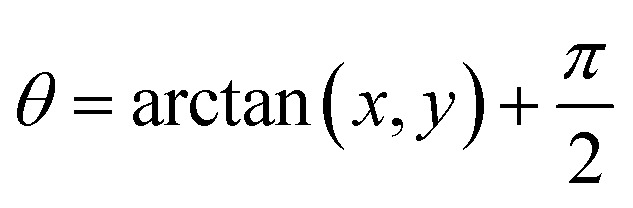
 to create defects with azimuthal orientation.

The value of the elastic Poisson ratio for rubber is 0.5, however, in order to avoid computational instability, we used 0.49. Using a shear modulus of *μ* = 5.7 × 10^5^ Pa, we obtain the value of Young's modulus *E* = 2*μ*(1 + *ν*) ≈ 1.7 × 10^6^ Pa, which is in the range of rubber elasticity. For simplicity, we set *α* = *μ* and Δ*S* = −1 so that the dimensionless parameter 
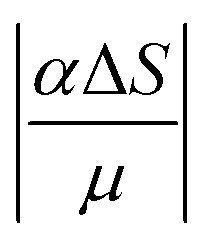
 which is responsible for the amount of deformation in the model equal to 1. We also normalize the deformations by their full range once fully actuated, such that +1 corresponds to the maximum elevation and 0 corresponds to the lowest value of deformations at indents.

## Author contributions

Yuxin You and Youssef M. Golestani (data curation; formal analysis; investigation; methodology; validation; visualization; writing original draft; and writing review and editing); Dirk J. Broer (conceptualization; formal analysis); Tinghong Yang (methodology) Guofu Zhou (funding acquisition; conceptualization); Robin L.B. Selinger, Dong Yuan and Danqing Liu (conceptualization; funding acquisition; project administration; supervision; and writing review and editing).

## Conflicts of interest

There are no conflicts to declare.

## Supplementary Material

MH-011-D4MH00131A-s001

MH-011-D4MH00131A-s002

MH-011-D4MH00131A-s003

## References

[cit1] Tramacere F., Beccai L., Kuba M., Gozzi A., Bifone A., Mazzolai B. (2013). PLoS One.

[cit2] Allen J. J., Bell G. R. R., Kuzirian A. M., Velankar S. S., Hanlon R. T. (2014). J. Morphol..

[cit3] Reiter S., Hülsdunk P., Woo T., Lauterbach M. A., Eberle J. S., Akay L. A., Longo A., Meier-Credo J., Kretschmer F., Langer J. D., Kaschube M., Laurent G. (2018). Nature.

[cit4] Huang G., Li S., Jin X., Qi M., Gong X., Zhang G. (2020). Colloids Surf., B.

[cit5] Kelman E. J., Osorio D., Baddeley R. J. (2008). J. Exp. Biol..

[cit6] Gonzalez-Bellido P. T., Scaros A. T., Hanlon R. T., Wardill T. J. (2018). iScience.

[cit7] Yang H., Buguin A., Taulemesse J.-M., Kaneko K., Méry S., Bergeret A., Keller P. (2009). J. Am. Chem. Soc..

[cit8] Agrawal A., Luchette P., Palffy-Muhoray P., Biswal S. L., Chapman W. G., Verduzco R. (2012). Soft Matter.

[cit9] Wang M., Chu D., Liu L., Huang S., Chen X.-M., Liu Z.-Y., Yang H. (2021). Chin. J. Polym. Sci..

[cit10] Liu D., Bastiaansen C. W. M., den Toonder J. M. J., Broer D. J. (2012). Angew. Chem., Int. Ed..

[cit11] Hendrikx M., Sırma B., Schenning A. P. H. J., Liu D., Broer D. J. (2018). Adv. Mater. Interfaces.

[cit12] Molina M. A., Rivarola C. R., Broglia M. F., Acevedo D. F., Barbero C. A. (2012). Soft Matter.

[cit13] Shahsavan H., Yu L., Jákli A., Zhao B. (2017). Soft Matter.

[cit14] Ji Y., Yang B., Cai F., Yu H. (2022). Macromol. Chem. Phys..

[cit15] Xin H., Chen H., Song P., Sun Q. (2023). Mater. Today Commun..

[cit16] Collyer A. A. , Liquid Crystal Polymers: From Structures to Applications, Springer Science, Dordrecht, 1993

[cit17] Ube T., Ikeda T. (2014). Angew. Chem., Int. Ed..

[cit18] Kuenstler A. S., Chen Y., Bui P., Kim H., DeSimone A., Jin L., Hayward R. C. (2020). Adv. Mater..

[cit19] Pilz da Cunha M., Debije M. G., Schenning A. P. H. J. (2020). Chem. Soc. Rev..

[cit20] White T. J., Broer D. J. (2015). Nat. Mater..

[cit21] Broer S. , Crawford D. and Zumer G. P., Cross-linked liquid crystalline systems: from rigid polymer networks to elastomers, CRC press, 2011

[cit22] Liu D., Broer D. J. (2015). Nat. Commun..

[cit23] Feng W., Broer D. J., Liu D. (2018). Adv. Mater..

[cit24] Feng W., Chu L., de Rooij M. B., Liu D., Broer D. J. (2021). Adv. Sci..

[cit25] Babakhanova G., Turiv T., Guo Y., Hendrikx M., Wei Q.-H., Schenning A. P. H. J., Broer D. J., Lavrentovich O. D. (2018). Nat. Commun..

[cit26] You R., Kang S., Lee C., Jeon J., Wie J. J., Kim T. S., Yoon D. K. (2021). ACS Appl. Mater. Interfaces.

[cit27] Zhang P., Kragt A. J. J., Schenning A. P. H. J., de Haan L. T., Zhou G. (2018). J. Mater. Chem. C.

[cit28] Ware T. H., McConney M. E., Wie J. J., Tondiglia V. P., White T. J. (2015). Science.

[cit29] Herbert K. M., Fowler H. E., McCracken J. M., Schlafmann K. R., Koch J. A., White T. J. (2021). Nat. Rev. Mater..

[cit30] Saeed M. H., Choi M.-Y., Kim K., Lee J.-H., Kim K., Kim D., Kim S.-U., Kim H., Ahn S., Lan R., Na J.-H. (2023). ACS Appl. Mater. Interfaces.

[cit31] Dey S., Agra-Kooijman D., Ren W., McMullan P., Griffin A., Kumar S. (2013). Crystals.

[cit32] Yu H. , Dancing with light: advances in photofunctional liquid-crystalline materials, Pan Stanford, Singapore, 2015

[cit33] Barrett C. J., Mamiya J., Yager K. G., Ikeda T. (2007). Soft Matter.

[cit34] Wang L., Li Q. (2018). Chem. Soc. Rev..

[cit35] Yu H., Ikeda T. (2011). Adv. Mater..

[cit36] Ikeda T., Mamiya J., Yu Y. (2007). Angew. Chem., Int. Ed..

[cit37] Chen Y., Yang J., Zhang X., Feng Y., Zeng H., Wang L., Feng W. (2021). Mater. Horiz..

[cit38] Song C., Zhang Y., Bao J., Wang Z., Zhang L., Sun J., Lan R., Yu Z., Zhu S., Yang H. (2023). Adv. Funct. Mater..

[cit39] Jiang M., Guo Y., Selinger R. L. B., Lavrentovich O. D., Wei Q. H. (2023). Liq. Cryst..

[cit40] Guo Y., Jiang M., Afghah S., Peng C., Selinger R. L. B., Lavrentovich O. D., Wei Q. H. (2021). Adv. Opt. Mater..

[cit41] Wu H., Hu W., Hu H., Lin X., Zhu G., Choi J.-W., Chigrinov V., Lu Y. (2012). Opt. Express.

[cit42] Lubensky T. C., Mukhopadhyay R., Radzihovsky L., Xing X. (2002). Phys. Rev. E: Stat., Nonlinear, Soft Matter Phys..

[cit43] Mark J. E. (1981). J. Chem. Educ..

[cit44] Clarke S. M., Tajbakhsh A. R., Terentjev E. M., Remillat C., Tomlinson G. R., House J. R. (2001). J. Appl. Phys..

[cit45] Jull E. I. L., Mandle R. J., Raistrick T., Zhang Z., Hine P. J., Gleeson H. F. (2022). Macromolecules.

[cit46] Obilor A. F., Pacella M., Wilson A., Silberschmidt V. V. (2022). Int. J. Adv. Manuf. Technol..

[cit47] Godman N. P., Kowalski B. A., Auguste A. D., Koerner H., White T. J. (2017). ACS Macro Lett..

[cit48] Zhan Y., Calierno S., Peixoto J., Mitzer L., Broer D. J., Liu D. (2022). Angew. Chem., Int. Ed..

[cit49] Geuzaine C., Remacle J. (2009). Int. J. Numer. Methods Eng..

[cit50] Gelebart A. H., Jan Mulder D., Varga M., Konya A., Vantomme G., Meijer E. W., Selinger R. L. B., Broer D. J. (2017). Nature.

