# Correction: Transforming patterned defects into dynamic poly-regional topographies in liquid crystal oligomers

**DOI:** 10.1039/d5mh90044a

**Published:** 2025-04-22

**Authors:** Yuxin You, Youssef M. Golestani, Dirk J. Broer, Tinghong Yang, Guofu Zhou, Robin L. B. Selinger, Dong Yuan, Danqing Liu

**Affiliations:** a Joint Research Lab of Devices Integrated Responsive Materials (DIRM), South China Normal University Guangzhou 510006 China dong.yuan@guohua-oet.com; b Human Interactive Materials (HIM), Department of Chemical Engineering and Chemistry, Eindhoven University of Technology Groene Loper 3 Eindhoven 5612AE The Netherlands d.liu1@tue.nl; c Institute for Complex Molecular Systems (ICMS), Eindhoven University of Technology Groene Loper 3 Eindhoven 5612AE The Netherlands; d Department of Physics, Kent State University Kent OH 44242 USA rselinge@kent.edu; e Advanced Materials and Liquid Crystal Institute, Kent State University Kent OH 44242 USA

## Abstract

Correction for ‘Transforming patterned defects into dynamic poly-regional topographies in liquid crystal oligomers’ by Yuxin You *et al.*, *Mater. Horiz.*, 2024, **11**, 3178–3186, https://doi.org/10.1039/D4MH00131A.

The authors regret the omission of the following funding information from the acknowledgments in the published article: Dutch Research Council (NWO) (OTP19440, OTP19966).

The correct acknowledgements section of the article reads as follows:

National Key R&D Program of China (2020YFE0100200), Guangzhou Science and Technology Project (No. 202201010351), Guangdong Provincial Key Laboratory of Optical Information Materials and Technology (No. 2017B030301007), National Center for International Research on Green Optoelectronics (No. 2016B01018), MOE International Laboratory for Optical Information Technologies and the 111 Project, the Natural Science Foundation of Guangdong Province (No. 2024A1515011829). Yuxin You is financially supported by the China Scholarship Council (CSC). This research forms part of the research program financed by the Dutch Research Council (NWO) (OCENW.KLEIN. 10854, START-UP 8872, Gravity Program 024.005.020 – Interactive Polymer Materials IPM, OTP19440 and OTP19966). This work was supported in part by an allocation of computing time from the Ohio Supercomputer Center. RLBS and YMG were supported by funding from NSF CMMI-1663041 for development of simulation methods.

The Royal Society of Chemistry apologises for these errors and any consequent inconvenience to authors and readers.

