# Supplementary material for: Transforming patterned defects into dynamic poly-regional topographies in liquid crystal oligomers
Source: Mater Horiz. 2024 Apr 19;11(13):3178–86. doi: 10.1039/d4mh00131a (PMC11216033; doi:10.1039/d4mh00131a)
Supplement: MH-011-D4MH00131A-s001 [file MH-011-D4MH00131A-s001.pdf]

Electronic Supplementary Material (ESI) for Materials Horizons.
This journal is © The Royal Society of Chemistry 2024

Supporting Information

Transforming Patterned Defects into Dynamic Poly-Regional Topographies in Liquid Crystal Oligomers

Yuxin You,[abc] Youssef M. Golestani,[bc] Dirk J. Broer,[bc] Tinghong Yang,[a] Guofu Zhou,[a†] Robin L.B. Selinger,*[de] Dong Yuan*[a] and Danqing Liu*[bc]

[a]Joint Research Lab of Devices Integrated Responsive Materials (DIRM), South China Normal University, Guangzhou 510006, China. E-mail: dong.yuan@guohua-oet.com

[b]Human Interactive Materials (HIM), Department of Chemical Engineering and Chemistry, Eindhoven University of Technology, Eindhoven 5612AE, The Netherlands. E-mail: d.liu1@tue.nl

[c]Institute for Complex Molecular Systems (ICMS), Eindhoven University of Technology, Eindhoven 5612AE, The Netherlands

[d]Department of Physics, Kent State University, Kent, OH 44242, USA. E-mail: rselinge@kent.edu.

[e]Advanced Materials and Liquid Crystal Institute, Kent State University, Kent, OH 44242, USA

The Supplementary Information includes:

Supplementary Text

Figures S1-S9

Video S1-S2

References

We characterized the degree of oligomerization for main-chain liquid crystal oligomers with azobenzene (Azo) concentrations of 1 wt.%, 5 wt.%, 10 wt.%, and 15 wt.%. **Figure S1** shows the ^{1}H NMR spectrum of partial protons of those oligomers. The signals between 5.75 ppm and 6.45 ppm correspond to the six protons in the diacrylate end groups in the main-chain liquid crystal oligomers. The signals around 7.88 ppm match with the four aromatic protons of azobenzene. The signals around 8.15 ppm correspond to the four aromatic protons in the aromatic groups of RM82. In this study, we define the average degree of oligomerization (DO) as the number of mesogenic units per oligomer. Assuming the chain-extension reaction is complete, the expected DO of the oligomer is calculated using Equation (1). S_{Ac} and S_{Ar} are defined as follows: S_{Ac} is the integration value of all protons in the diacrylate end groups divided by three, while S_{Ar} is the integration value of all the aromatic protons in the mesogenic units. In each oligomer chain, there are three pairs of protons with identical chemical environments in the acrylate end groups, and the backbone contains 4*DO aromatic protons. When normalizing S_{Ac} to 2, S_{Ar} will adjust proportionally, simplifying Equation (1) to Equation (2).

$$\frac{S_{Ac}}{S_{Ar}} = \frac{2}{4 * DO} \tag{1}$$

$$DO = \frac{S_{Ar}}{4} \tag{2}$$

The average degrees of oligomerization of the main-chain oligomers were calculated as follows: DO (1 wt.% Azo) = (33.91 + 0.47) / 4 = **8.60**; DO (5 wt.% Azo) = (32.04 + 2.30) / 4 = **8.59**; DO (10 wt.% Azo) = (30.27 + 4.99) / 4 = **8.81**; DO (15 wt.% Azo) = (26.54 + 7.73) / 4 = **8.57**.

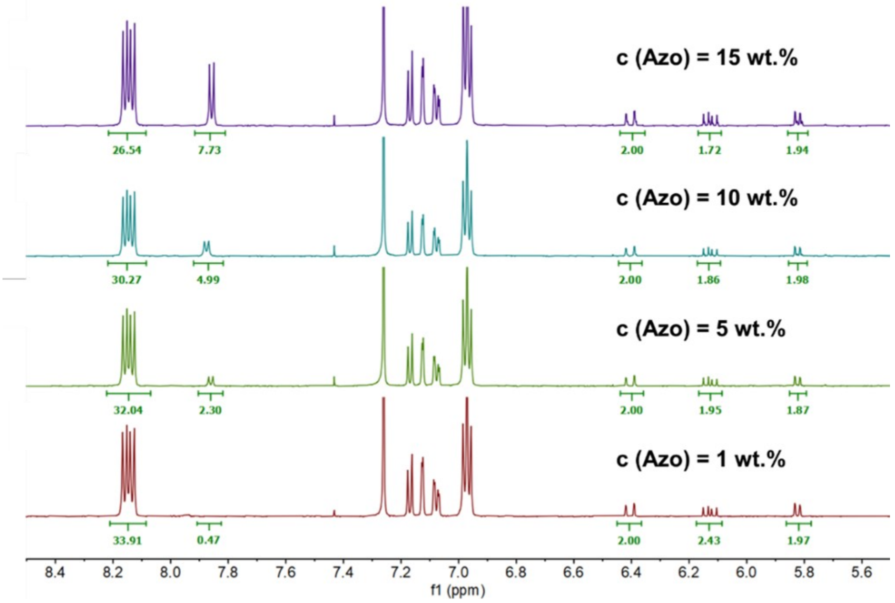

Figure S1. ^{1}H NMR spectrum of partial protons in main-chain Oligomers with azobenzene concentrations of 1 wt.%, 5 wt.%, 10 wt.%, and 15 wt.%.

We studied the phase behavior of the main-chain oligomers before crosslinking. As shown in **Figure S2**, the smectic to nematic transition occurs at 48 ºC during heating and cooling procedures. The nematic to isotropic transition occurs at 89 ºC, 86 ºC, 81 ºC, and 78 ºC for azobenzene concentrations of 1 wt.%, 5 wt.%, 10 wt.%, and 15 wt.%, respectively. Based on this measurement, we crosslinked the oligomers at the nematic temperature above 50 ºC.

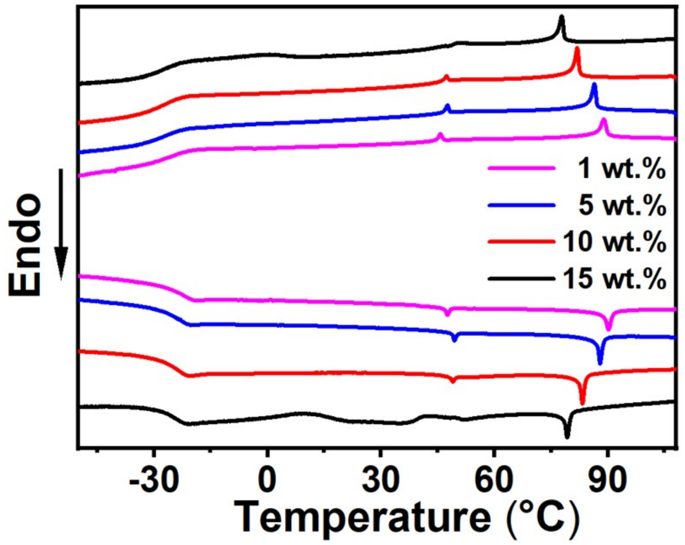

Figure S2. DSC analysis of liquid crystal oligomers with azobenzene concentrations of 1 wt.%, 5 wt.%, 10 wt.%, and 15 wt.%, respectively. The measurement was conducted at a ramping rate of 10 ºC min^{-1}.

We studied the glass transition temperature (T_g) of the liquid crystal oligomer networks. As shown in **Figure S3**, their glass transition temperatures occurred around -14 ºC, -15 ºC, -17 ºC, and -19 ºC for azobenzene concentrations of 1 wt.%, 5 wt.%, 10 wt.%, and 15 wt.%, respectively. These oligomer networks are in a rubbery state at room temperature.

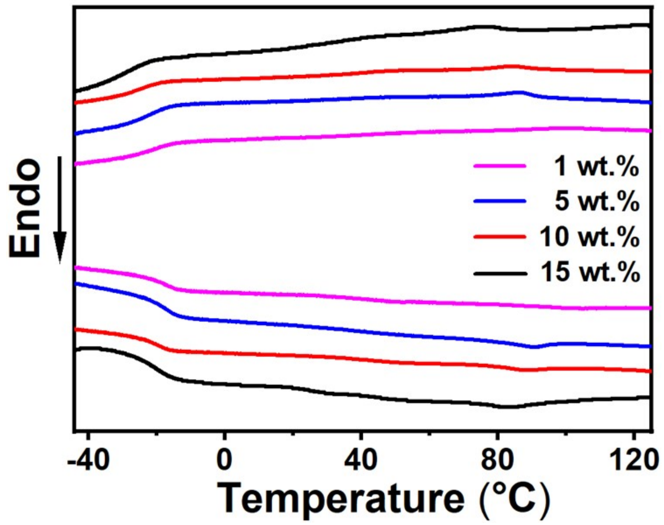

Figure S3. DSC analysis of liquid crystal oligomer networks with azobenzene concentrations of 1 wt.%, 5 wt.%, 10 wt.%, and 15 wt.%, respectively. The measurement was conducted at a ramping rate of 5 ºC min^{-1}.

We selected an LCON coating with a thickness of 4.9 µm and an azobenzene concentration of 15 wt.% to further study the permanent surface topographies. We observed that while continuously heating above 90 ºC, the surface started to undergo plastic deformation.[1] Typically, at 120 ºC, the deformation reached up to 3.89 µm (**Figure S4c**), with an amplitude of 80%. Upon cooling, the coating did not fully return to the initial flat state, retaining 28% of the deformation.

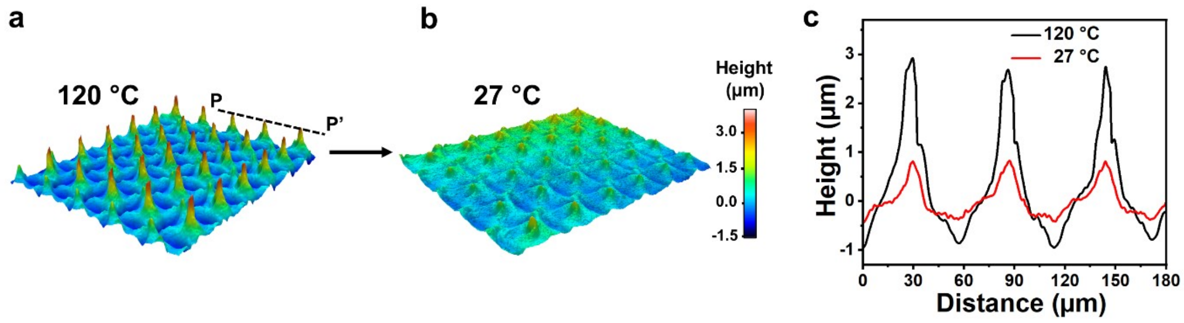

Figure S4. 3D images of an LCON coating with azobenzene concentration of 15 wt.% at a) 120 ºC and b) 27 ºC. c) Cross-section surface profiles along line **PP'**.

We varied the coating thickness of an LCON coating and we observed that the thicker coating deforms greater. This can be interpreted as increased thickness providing a larger reservoir of materials available for migration. On the other hand, in the context of the surface of the coating, the materials below the surface can be considered as the soft sublayer. When the thickness of the coating increases, it is equivalent to increasing the thickness of the soft sublayer, which contributes to enhancing the deformation amplitude of the surface topographies.[2]

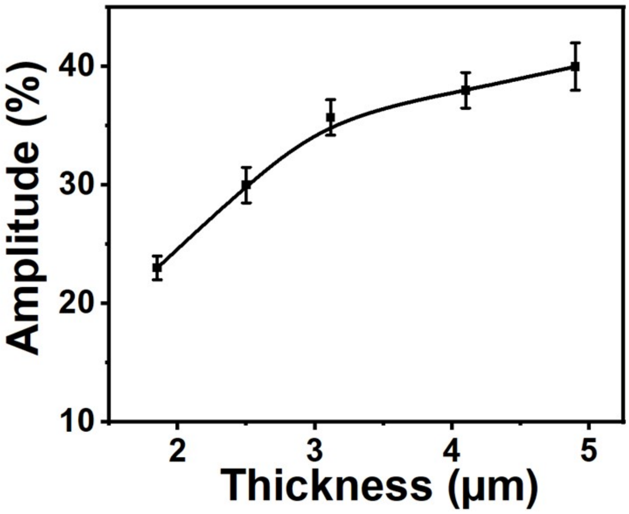

Figure S5. Relationship between thermal response amplitude and thickness of the coating. The sample measured contains 15 wt.% azobenzene.

To reveal the relationships between the deformation and coating thickness, we kept the UV light on and investigated the deformation of coatings with varying thicknesses at room temperature or 53 ºC, respectively. At room temperature, the increase in deformation with increasing thickness was not significant, while at 53 ºC, the deformations showed a notable increase, indicating a more significant synergistic effect. Typically, a coating with a thickness of 5 μm exhibits an amplitude of close to 60%.

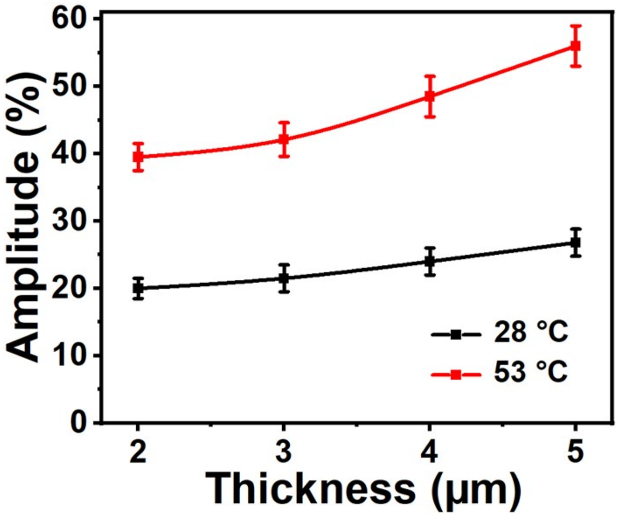

Figure S6. Influence of thickness on the deformation amplitude induced by light illumination at room temperature or 53 ºC, respectively. The sample contains 15 wt.% azobenzene.

Director field design in our model is a 3×3 array of +1 and -1 topological defects with azimuthal orientation and periodicity P = 60 μm. The defect lines extend from the bottom of the coating to the top and are oriented perpendicular to the rigid substrate. Each defect is located in the center of a square mosaic tile where the director at a given position (x,y) is $m \times \arctan(x,y) + \theta_0$. Here, m is the strength of the defect, $\arctan(x,y)$ is the spatial angle in polar coordinates, and θ_0 is a constant phase angle and is set to 90º to represent an azimuthal director field configuration. The regions identified in **Figure S7** follow the labeling of regions in the experiment.

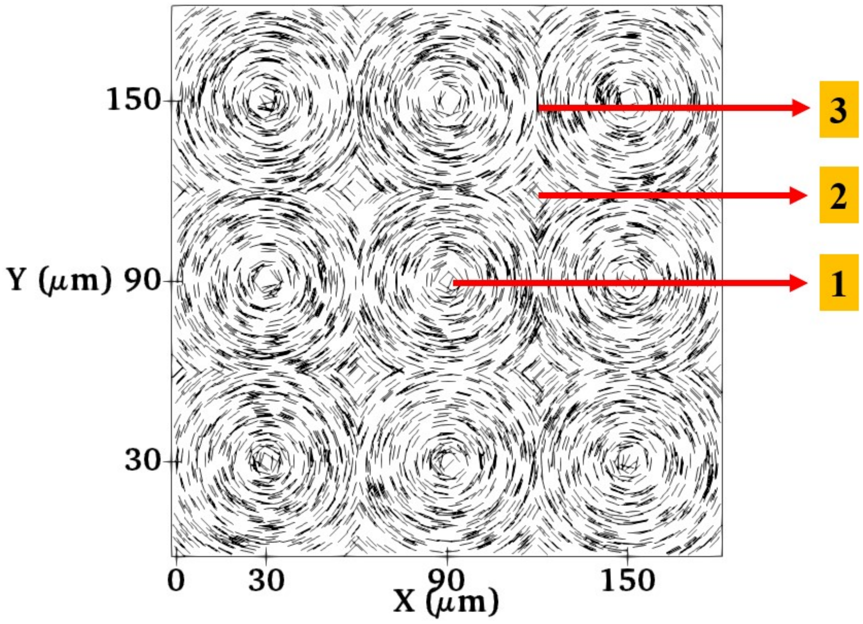

Figure S7. Director field design in the FEM elastodynamic simulation.

The corresponding surface topography of the coating, obtained from FEM simulation, is shown in **Figure S8**. The film thickness is 5μm and the amplitude of deformations grows uniformly in the sample as the scalar order parameter drops linearly. Images shown here are obtained by setting the dimensionless parameter $\frac{\alpha|\Delta S|}{\mu}$ equal to 1. The top and bottom images are shown in false (z axis has different metrics with respect to x and y), and true axis (all axes with the same metric), respectively.

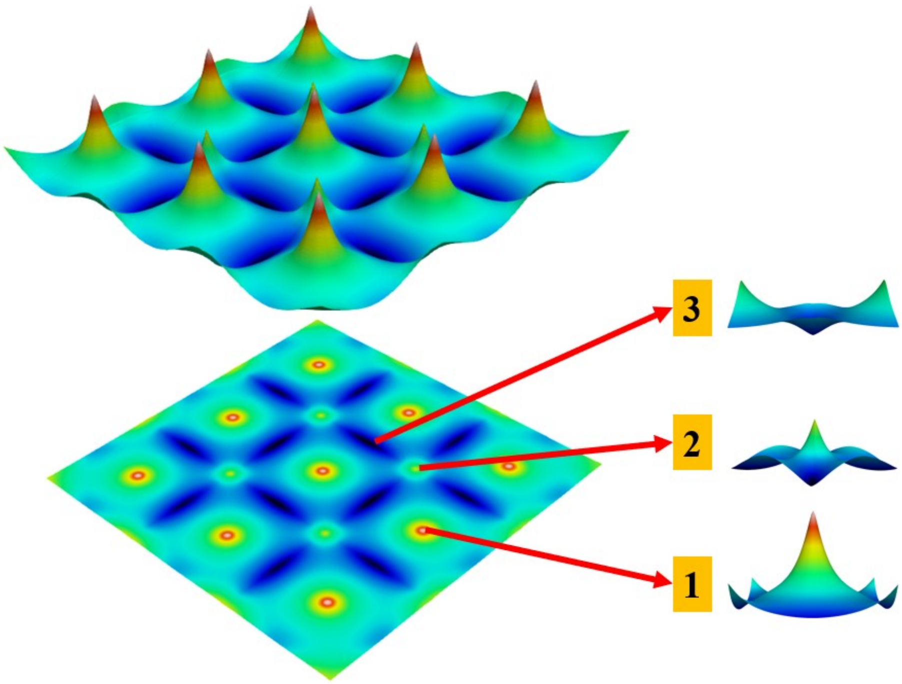

Figure S8. Simulation of surface topography for a coating with a 3×3 array of +1 and -1 topological defects. The onset shows the side view of the regions.

To study the contribution of spatial uniformity during actuation, we modify our FEM code by adding a spatial gradient to control the change in the scalar order parameter S along the thickness. To do so, we choose three functions: one linear and two exponential functions in the form of $e^{\frac{(z-d)}{L}} \times S_{z=d}^{(t)}$, where d is the thickness of the coating, L is the length scale related to the penetration of light absorption during actuation, and $S_{z=d}^{(t)}$ is the value of S at the top layer at a given time t (**Figure S9a)**. This way, the top layer of the sample undergoes full actuation, $\Delta S = -1$; while the bottom layer is either partially actuated or remains unactuated, i.e., $\Delta S = 0$ (in the case of linear gradient), see **Figure S9b**. We find out that, when there is a gradient, the amplitude of deformation on the surface of the sample is lower than the case of uniform actuation (**Figure S9c**). Consequently, we propose this as a possible reason for observing a lower amplitude of deformation by UV light actuation in our experiment. Although heat increases the temperature almost uniformly through the thickness of the coating, absorption by UV irradiation is significantly different on the top and bottom of the coating.

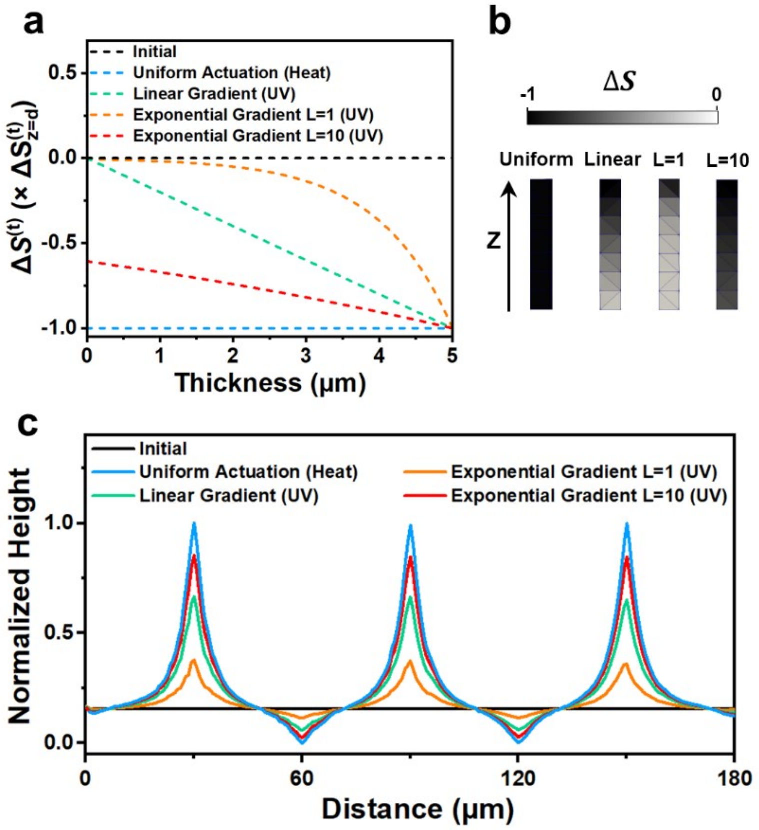

Figure S9. a) Proposed mathematical equations used to investigate the spatial dependency of the change in S. b) Color plots of S for each element across the thickness. The triangles show

the outer side of the tetrahedra. c) Cross-section surface profiles, obtained by running FEM simulation.

References

1 A. F. Obilor, M. Pacella, A. Wilson and V. V. Silberschmidt, *Int. J. Adv. Manuf. Technol.*, 2022, **120**, 103.

2 M. Hendrikx, B. Sırma, A. P. H. J. Schenning, D. Liu and D. J. Broer, *Adv. Mater. Interfaces*, 2018, **5**, 1.

Captions for Video S1 and S2

Video S1.

Digital holographic microscopy movie shows the reversible surface deformation upon light illumination at 53 ºC.

Video S2.

The video of the FEM simulation shows the shape transformation of the surface upon actuation and deactivation.
